# Experiences of patients with anorexia nervosa during the transition from child and adolescent mental health services to adult mental health services

**DOI:** 10.1186/s40337-020-00313-4

**Published:** 2020-08-11

**Authors:** Veronica Lockertsen, Liv Nilsen, Lill Ann Wellhaven Holm, Øyvind Rø, Linn May Burger, Jan Ivar Røssberg

**Affiliations:** 1 Division of Mental Health and Addiction, Oslo University Hospital, P.O. Box 4959, Nydalen, Oslo, Norway; 2 Institute of Clinical Medicine, Faculty of Medicine, University of Oslo, 0318 Oslo, Norway; 3 Oslo, Norway; 4 Regional Department for Eating Disorders, Division of Mental Health and Addiction, Oslo University Hospital, Ullevål HF, Postboks 4950 Nydalen, 0424 Oslo, Norway; 5 Drammen, Norway

**Keywords:** Anorexia nervosa, Mental health transition, Adolescent, Patients’ perspective, Qualitative research

## Abstract

**Background:**

The transition between the Child and Adolescent Mental Health Services (CAMHS) and the Adult Mental Health Services (AMHS) is identified as an especially critical time for the continuity of care for patients with anorexia nervosa (AN). However, research on this topic is scarce. In the present study, we explore the patients’ experiences of the transition between CAMHS and AMHS.

**Methods:**

A qualitative explorative study was carried out based on recorded interviews from one multi-step focus group and six individual interviews with patients who experienced the transition from CAMHS to AHMS in Norway. This study is service user-initiated, meaning service users were involved in all steps of the research process.

**Results:**

The adolescents’ experiences are characterized by four overall themes regarding the transition process between CAMHS and AMHS: (1) “Being unprepared and alone in the transition process” describes how a lack of preparation for the transition between CAMHS and AMHS makes them feel alone and increases stress. (2) “It takes time to create a trusting relationship” describes how time influences patients’ trust in therapists and motivation for treatment. (3) “We are not all the same” describes how adolescents develop differently but are not treated differently despite their diverse ability to be self-sufficient. (4) “How they see me and treat me affects my hope for the future” describes the interaction between adolescents and therapists.

**Conclusions:**

Acknowledging the patients’ needs during the transition period and considering their readiness for the transition is important. Taking into account the four dimensions described in the present study might improve the transition process and enhance the patients’ self-sufficiency and maturity.

## Plain English summary

Adolescents with anorexia nervosa (AN) who receive care from Child and Adolescent Mental Health Services (CAHMS) often need further treatment from Adult Mental Health Services (AHMS). This study explored the transition between CAHMS and AHMS for patients with AN in a naturalistic public mental health-care setting. Our study identified four themes concerning the transition between CAMHS and AMHS, which patients considered influenced their transition and which demonstrated their lack of preparedness for the transition: The patients often felt alone in this period; time was considered an important element in developing trust in the therapeutic relationship; having the transition adapted to their individual needs and developmental stage is important for the transition outcome; how they were introduced to their new therapists and treated had an effect on their hope for recovery.

## Introduction

As many patients experience the transition process between Child and Adolescent Mental Health Services (CAMHS) and Adult Mental Health Services (AMHS) as less than optimal, and many patients drop out of treatment [1], continuity of care in the transition from CAHMS to AMHS is of major importance. A qualitative meta-synthesis focusing on the transition of chronically ill adolescents found that adolescents are unprepared for the transition to AMHS and that the loss of a familiar surroundings and relationships they had at CAMHS creates anxiety [2]. Adolescents are vulnerable to the changes that occur during the transition process, and the inflexibility of the service systems puts many adolescents at risk of disengaging from further treatment [1]. Less than 5% of 154 adolescents with a mental health disorder experienced a satisfactory transition [3]. Research show that several mental health care systems, do not have satisfactory cooperation between the CAMHS and AMHS, which creates a lack of continuity for mental health patients [4, 5]. It is thus recommended that the transition process from CAMHS to AMHS should be carefully planned, taking into consideration the medical, psychosocial, and educational/vocational needs of the adolescents [6, 7]. The patients should be properly engaged at AMHS before their therapy at CAMHS is terminated, and they should view the transition period as a time to practice self-sufficiency and increase empowerment. A strong focus on family involvement in the transition period, is important [8, 9]. Anorexia nervosa (AN) is a severe psychiatric disorder that patients often struggle with for several years. The disorder often results in severe psychosocial and physical consequences for the individual and their family. Age of onset is typically between 14 and 19 years [10], and approximately 85% of the cases begin before the age of 20 [11]. Additionally, patients with AN often have comorbid disorders, such as substance abuse, depression, obsessive-compulsive disorder (OCD), anxiety, and post traumatic stress disorder (PTSD), which complicate the recovery process [12, 13]. Patients with AN often have a devastating fear of gaining weight; the nature of an eating disorder often implies that the patient does not consider themselves ill and has poor insights into the consequences of the illness, which frequently creates a struggle with motivation for recovery [14]. Patients claim that AN gives them a feeling of control and protection, making it difficult for them to let go of the disease and thus comply with treatment. As a result of the above, patients with AN often have complex needs in the transition period, and a thoughtful transition plan is necessary to maintain treatment progress [15]. Although there are variations between different local treatment cultures, CAMHS and AMHS have quite different approaches regarding the extent to which they engage the parents in the treatment [16]. The treatment approach at AMHS is based on individual responsibility. Dimitropoulos, Toulany [17] questioned these sudden changes in treatment approaches to determine whether the supporting surroundings at CAMHS offer adolescents the opportunities they need to practice self-sufficiency and eat independently without familial support. As patients with AN are thought to lag behind their peers both developmentally and psychologically, they typically require careful preparation to manage the transition process [18]. Professionals have underlined how a barrier is formed in the transition when age, rather than readiness, is set as a marker for timing the transition [18]. Most high-income countries divide their services between CAMHS and AMHS at around age 18 [19].

To increase the possibility of adolescents managing the transition, it is essential to have good collaboration between the two services. Nevertheless, a previous study found that the current lack of cooperation between CAMHS and AMHS makes it difficult for patients to navigate between the two services [20]. This gap seem to be present in several countries, regardless of how CAMHS and AMHS are organized [21]. There have been an increasingly awareness of the challenge’s patients have in the transition process between CAMHS and AMHS. Based on equal input from relatives, professionals, and the patients, the Canadian Eating Disorder Priority Setting Partnership has prioritized research topics for female patients with AN [22]; they identified that one of the top priorities relates to gaps in treatment, suggesting that research should focus on the transition between services. “Integrated care” have also been identified by clinicians and service users, as a research priority in an Australian setting [23]. Although previous studies have investigated relatives’, professionals’, and patients’ experiences of transitions in specialized eating disorder programs [17, 18, 24], few have focused solely on the patients’ perspectives during this transition period, when treated in a naturalist public mental health-care setting. A greater understanding of how patients experience this transition through the different levels of organized health care could contribute to a more cohesive treatment process for patients. Thus, we interviewed patients with AN to understand their experiences of the transition from CAMHS to AMHS.

## Methods

### Design

The current study is part of a qualitative research project designed to investigate the experiences of the transition between CAMHS and AMHS for patients, parents, and professionals called Mind the Gap. To investigate the transition between CAMHS and AMHS from different perspectives, we used qualitative techniques, including semi-structured interviews and multi-step focus groups [25]. Mind the Gap is a user-initiated project, which implies that the experiences from a support group for relatives form the basis of the research questions. In the current study, the involvement of service users have influenced the research process from the start (designing the study) to the final version of the present manuscript. A former service user who has experience with AN (LMB) participated in the design of a semi-structured interview guide and moderated the interview in collaboration with the first author (VL). The research group also included a former service user with experience as a relative to a patient with AN (LAWH), two RN nurses (LN, VL), and two psychiatrists (JIR, ØR). We found this multi-perspective research group effective for forming the research questions, designing the interview guide, preparing and conducting the focus group interviews, and analyzing the transcriptions.

### Recruitment and participants

A total of 10 participants were included in the study. All participants had experiences with the transition between CAMHS and AMHS in a specialized mental health care services. Six had experiences from inpatient units and three had been treated in a specialized eating disorder unit in CAMHS. Five of the participants had been treated in a specialized eating disorder unit in AMHS. All participants were women, with a median age of 22 (range; 19–29) years. The median time since the transition between CAMHS and AMHS was 4.5 years (range; 1–10 years). Six of the participants were working or studying at the time of the qualitative interview. Eight of the participants still received treatment. Six of the participants were invited to participate by current therapists who contacted participants they knew had experiences that could illuminate the research questions. The others made contact with the project manager after finding project information on an internet support site for eating disorders. Recruitment took place between February 2018 and May 2019. Written consent was obtained from all participants.

### Data collection

Semi-structured interviews (lasting 60–90 min) were conducted by the fifth (LMB) and first (VL) authors as moderator and co-moderator, respectively. In addition to former user experiences, LMB has attended service user boards in hospitals and performed voluntary work in an interest group for eating disorders. We conducted five individual interviews and one multi-step focus group interview with four participants to explore the experiences of AN patients in the transition between CAMHS and AMHS. The individual and focus group interviews were both guided by our research question, and participants were encouraged to provide concrete examples of their experiences. Focus group interviews are described as a suitable technique in qualitative research because they elicit people’s understandings and views of their experiences [26]. Focus groups can be stimulating environments, with co-participants positively triggering other participants’ memories. Multi-step focus groups are considered a beneficial method when working with service users in research [27]. Repeated meetings give the participants an opportunity to inspect and challenge their own and others’ experiences in a dialectic way. Between the focus group meetings, we handed out a summary of the first focus group interview, so the participants could prepare for the second meeting. This summary constructed the main themes we wanted to focus on in the second group meeting. The individual interviews supplied an in-depth answer of the research question. The first author recorded and transcribed the interviews verbatim. The interviews were conducted at the research clinic or in other neutral settings.

### Data analysis

Data were analyzed using a systematic text condensation inspired by Giorgi [28, 29]. The method focused on presenting the patients’ expressed experiences, rather than a possible underlying meaning of what was said. Using a method of de-contextualization and re-contextualization of the text, we analytically reduced the data and created a condensed text that represented the overall material [28]. The process comprised four steps: In the first step, the research group read the interviews several times to form an overall impression of the material. In step two, we started sorting the text. Text that belonged together was grouped and encoded. The codes were adjusted and organized so that themes with common content were collected under the same code. In step three, the meaning contents of the different codes were abstracted and collected across the interviews. Step four is the analytical text that represents the results of the research project. With our co-operative relationship, we managed to have a multi-perspective view of the data. To help organize and categorize the transcriptions, we used the computerized program NVivo 11 (QSR International Pty Ltd.)

### Ethical aspects

The study was approved by the data protection authority at Oslo University Hospital OUS (2016/19732) and performed in accordance with the Declaration of Helsinki. The Regional Committee for Medical and Health Research Ethics for the Southeast Region of Norway (2016/1259) viewed the study not to be a medical or health-related research project regulated by the law of health research and was not therefore subject to presentation, cf. HFL. § 2.

## Results

The participants’ experiences are characterized by four overall themes regarding the transition process between CAMHS and AMHS: (1) “Being unprepared and alone in the transition process” describes how a lack of preparation for the transition between CAMHS and AMHS makes them feel alone and increases stress. (2) “It takes time to create a trusting relationship” describes how time influences patients’ trust in therapists and motivation for treatment. (3) “We are not all the same” describes how participants develop differently but are not treated differently despite their diverse ability to be self-sufficient. (4) “How they see me and treat me affects my hope for the future” describes the interaction between participants and therapists.

### Being unprepared and alone in the transition process

The participants described a feeling of being unprepared and alone during the transition between CAMHS and AMHS. Some patients described that their Primary Care Doctor (PCP) tried to compensate for the lack of follow-up by offering ad hoc support: “I went so many months on my own—without professional contact, just with a support-call from my PCP doctor.” They felt overwhelmed when entering AMHS and were unprepared to handle the differences between CAMHS and AMHS. When treated at CAMHS, they felt they were followed up more closely during unstable periods. However, at AMHS, they were often discharged: “Here I got discharged when I was too ill to manage the treatment program.” The participants believed they would feel less alone and stressed if CAMHS had a more active function in the transition process. “I remember my mom called CAMHS to ask if I could come back, just after turning 18, but they turned us down.” Some participants explained that their parents became increasingly discouraged because they felt as if they had nowhere to turn to for help: “My parents lost hope after CAMHS because they thought that CAMHS had tried everything and that we had nowhere to turn to. So, my parents believed that this would last for the rest of our lives.” Often when the participants felt alone and scared during the transition process, they turned to and focused more on their eating disorder to gain a sense of control in the situation. However, focusing on their AN ameliorated their internal chaos. Most of the participants said they were unprepared for the expectation at AMHS for them to be self-sufficient and for how their parent’s role changed during the transition phase: “I went from being admitted with my parents in CAMHS to being transferred to a ward that treated my parents as distant relatives, with restrictions about when to call them and what to let them know.” It was new for the patients to be responsible for passing information forward and managing the collaboration with their parents. Following the active role their parents had played in CAMHS, some participants felt it important to establish themselves as independent and maintain control. Therefore, many participants found it difficult to uphold an open dialog with their parents during the transition period. Differences in how parents were included in the treatment between CAMHS and AMHS made the patients concerned about their parents’ well-being. They considered it important that their parents also received support and help from the therapists at AMHS.

### It takes time to create a trusting relationship

Overall, the participants viewed their first meeting at AMHS as important for their adherence to the treatment. The participants often described themselves as being insecure when establishing a relationship at AMHS. They felt they were met with a preconception of what they were, and they were afraid that the new therapists would be unable to understand and acknowledge their situation. The participants were worried about being misinterpreted and were concerned that their story could turn into something they did not recognize as their own. Consequently, they held back, but they acknowledged that doing so made it difficult for the new therapist to help them. The participants stated that it would have been easier for them to connect if CAMHS had prepared them for the transition. Some participants believed that it would be easier to communicate with their new therapist at AMHS if their CAMHS therapist had been present for support in the first meetings. As one adolescent said, “I got scared in the first meeting because I was used to a warmer, more caring, and humble approach. At CAMHS, they often sat beside me, but at AMHS, I sat on the opposite side of a large table with a therapist that did not say much.” When establishing a new relationship, the participants needed time to open up about their weight and food issues. They expected the therapists to understand this need, so it was difficult for the participants to understand why they rushed them into gaining weight, and why they—so early in the relationship—gave the responsibility for recovery back to the patients. They felt a need for more structure and time to feel safe and ready to work on their problems regarding food and their emotions. The participants often described how difficult it was to engage in treatment when entering AMHS. They mostly explained this in terms of how the treatment was carried out at CAMHS and how it ended. As they were defined as adults when they turned 18, CAMHS terminated the therapy. While some understood why the transition was timed as it was and felt included and ready for a change; others felt the timing to be arbitrary and felt ignored in the transition process. This made them feel let down by CAMHS, which led some to mistrust towards the treatment system.

The participants felt that the therapists failed to understand what state they were in and how long it could take to build a satisfactory relationship between them and the therapists; they expected too much, too fast. For the participants, this created an issue of mistrust and allowed room for misunderstandings between the therapist and patients: “Perhaps she just wanted to tell me that this is going to be all right, but that was not the way I understood it.”

During the transition process to AMHS, the patients described many insecurities about who they were going to meet and how they would be met. They described how they were moved from person to person and how they were expected to follow and trust each new person they met. This was challenging for the participants because the information between CAMHS and AMHS was lacking, and the participants often had to repeat themselves, which made it difficult for the participants to develop motivation for their recovery. However, they managed to gain weight just by doing what they were told: “I realized that I needed the help—but I was never safe enough to choose to stay voluntary.” The participants said it was important to have a therapist who showed that they cared about them and their problems. To find a way to trust their therapist, the participants described different ways to test the therapists’ persistence and motivation. It could be as specific as in a meal situation or in evaluating the therapists’ responses in conversations, e.g., what words they used. If therapists showed that they cared, and “passed their tests,” the patients described that the motivation for recovery often increased, which gave them hope:

It was really important for me to have them close and see their engagement. That was the first time I had my own desire for recovery. You get so tired of the fight between your own two voices that it has to be the people meeting you that figure it out.

The participants described how much easier it was to trust therapists that seemed to be transparent. Some of the patients had experiences with therapists at CAMHS who they felt misused their trust by hiding information from them and going behind their backs to talk to their parents. When they had met a therapist had helped them, they found that they were often the ones who had spent the most time with them and were authentic:

It takes more time than a biweekly session. Now is the first time I have had a relationship with a therapist in almost a year. It is working now because we have passed the point of meeting each other with our assumptions, and I know he knows me—for me. I can be honest, and he knows my little signs of hurting.

### We are not all the same

Some of the participants felt ready for the responsibility that AMHS expected of them, which was to based on AMHS’s more individual, independent approach. Others found this shift in approach from that used at CAMHS too demanding. The participants missed being met with curiosity about what they viewed best for themselves during the transition process, including their ideas about how to recover. Despite having the same diagnosis, the participants were in different developmental stages and thus had different needs during the transition: “I was not ready—they wrote from CAMHS that I was eighteen on paper, but my mind was fourteen.” The participants described a sense of lagging behind their peers because they had been ill for many years. Some participants explained that they felt almost developmentally disturbed because the years during which they lacked normal contact with school and friends had had a fundamental effect on their development and social skills. It can be painful being defined as an adult when you feel much younger. They felt that the mental health system has a rigid understanding of what an adult is, being defined by the age boundary of 18: “They have to look into the situation and the person. We are not the same.” The participants agreed that the transition should be timed based on their developmental stage rather than their age.

While some described the transition to AMHS as something that prolonged their recovery process, others described it as vital for their improvement. They claimed that they were somewhat tired of CAMHS’s more childish approach and were ready for the transfer at the age of 17. They felt they were emerging into adulthood and were treated with too much caution at CAMHS: “I had to solve some problems myself and could not depend on other grownups. I had to be a person in my own life.” Although some of the participants had negative experiences of the transition, they thought it was important for their recovery process because it underlined the changes they had to make in regard to how they take responsibility. They claimed that “the transition can help reinforce that you are the most important person in your life and the only one who can change something. You’re your mom or your therapist.” They began to realize that they had to change, take control of their life, and become accountable to themselves, but they had to be ready to do so.

### How they see me and treat me affects my hope for the future

The participants often underlined that they felt powerless during the transition; they were dependent on how the system and professionals related to them and perceived them during the transition process. These interactions not only had a practical meaning, but they were also deeply important to the participants. The participants described how the choice of words, spoken or written, influenced their motivation for recovery: “When you are met with ‘this is chronic’ at the age of 17, what is the point of trying? This just confirms that it is hopeless.” For the adolescent patient, it was difficult to maintain motivation when they sensed that their surroundings did not envision their recovery. According to the participants, the difference between CAMHS and AMHS was evident in how they verbalized their visions and hope for the adolescent patient. While CAMHS focused on their future and the life they had in front of them, giving them hope, AMHS did not have the same focus in, for example, how they facilitated maintaining obligations to school or work. The participants perceived an attitude at AMHS that suggested they had to figure those things out on their own. Some participants were transferred from CAMHS to an adult acute psychiatric mental health ward, which they found particularly challenging. They described their mental state as being in crisis and could not understand why CAMHS refused to prolong their treatment. They thought that AMHS did not understand how frightening it could be to be admitted in an adult environment:

I had really nothing to say in that decision; they just let me know that I was going. I stayed for 8 months. I do not think that a 17–18-year-old is suited to an adult acute mental health care unit.”

The powerlessness they felt during the transition underlined feelings of aloneness and being unvalued. They describe a sense of resignation—“so I lost interest”—mostly because they felt AMHS lacked an understanding of the difficulties they were experiencing during the transition period.

When treated in in-patient care, they were admitted with other patients who had been ill for a long time. These factors influenced the participants’ hope for their recovery in the future: “At AMHS, I was admitted with patients that had been ill longer than I had been living.” They described a system that met them with phrases like “early disability benefits,” and their illness was described as “treatment resistant.” They explained how such words affected their views of their future because such words would stick, influencing how the rest of the treatment system viewed them and contributing to misconceptions, which narrowed their room to change. As a result, the participants became more dependent on their relationships outside of the treatment system to maintain hope for them, as they found it challenging on their own.

When entering the transition phase, the participants often felt as though AMHS had more interest in their illness than in them. The therapist seemed to place more importance on evaluating and interpreting their symptoms and BMI than talking with them about their well-being and experiences of their condition. Some stated that everything in therapy at AMHS was oriented around food and weight, which made it difficult to create trusting relationships with the therapists, who interpreted their BMI as mental and emotional problems. There was a high focus on their somatic health, but little interest in their mental health: “If you eat your food, then you are OK. If your BMI is close to normal, you are good.” They were asked if they wanted to have their parents with them, but when they responded negatively, they were never asked “why not.” The participants felt that therapists at CAMHS seemed more interested in getting to know them as a person, while those at AMHS only wanted to understand their disease: “You become your symptoms and they just ask: how can we medicate that?” With the experienced alteration from being treated as an individual to being treated as an illness, the participants often felt like an object during the transition, dependent wholly on a relationship that was restricted more to the registration of symptoms and less to what they felt would help them. They verbalized a sense of no longer feeling like a human, just classified symptoms with associated expectations about what they should be struggling with. In their adolescent years, many of them only had social contact with people who were getting paid to be there. They had to protect themselves from becoming attached to the people surrounding them, which they felt changed how they related to people in their lives outside the mental health services. For some, that situation magnified the symptoms of the AN because of the safety they represented:

In the beginning, I got really attached to people, and when they got replaced, I was really affected. But now, it affects me less and less, and the AN has become more stable and safer—and it is always there for me.

## Discussion

The findings of the current study provide a meaningful contribution to the research, which explores the experiences of participants with AN during the transition between CAMHS and AMHS. The qualitative analysis revealed four overall themes regarding the transition process: (1) “Being unprepared and alone in the transition process,” (2) “It takes time to create a trusting relationship,” (3) “We are not all the same,” and (4) “How they see me and treat me affects my hope for the future.” The themes are interrelated and display elements with the transition that positively and negatively influence the participants’ experiences. These essential themes are discussed in the following paragraphs.

### Being unprepared and alone in the transition process

The current study found that the participants were unprepared for the transition to AMHS, which led to increased feelings of being alone. Without an understanding of the differences in the treatment approaches between the services, they did not feel as though they were taken seriously at AMHS. They felt alone and without what they considered adequate support during the transition phase. Our study supports the findings of Newlove-Delgado, Ford [30], who found that participants with ADHD were unprepared and left alone during the transition. As in our study, only one of the seven participants included in the study experienced to be prepared for the transition. Good preparation was followed by less stress and fewer feelings of uncertainty. Exploring the transition from the perspectives of 21 participants treated by the mental health services, Cleverley, Lenters [31] described the transition as “objectively terrifying” and the lack of preparation as “overwhelmingly present.” In the current study, it is unclear whether the experienced cultural and philosophical differences in approaches, was uncommunicated or not understood by the participants. Nonetheless, Winston, Paul [16] underlined the importance of discussing the differences between the services explicitly, with both the participants and their families. Consequently, the participants in current study lacked the ability to handle the sudden expectations of autonomy. Their destructive eating routine increased when in need for a feeling of safety they felt the illness (AN) provided them when the services did not. When exploring the transition of the patients with AN from the therapists’ perspectives, Dimitropoulos, Tran [14] emphasized that for patients with AN, preparing for the transition is particularly important because they often display less self-sufficiency and have difficulties fully engaging in treatment. Similar to our study, Dimitropoulos, Toulany [17] found that it would be beneficial for the patients and their relatives to have a clearer understanding of the implications of the treatment methods used in AMHS. Since patients often are dependent on parents’ external support in this phase, Dimitropoulos, Tran [14] underlined the importance of relatives being involved in the process. It could be argued that with more information and better preparation, parents would be better able to provide the support participants need during the transition process. As such, adequate support and preparation from the care system could decrease the patient’s feelings of being alone during this time. Ideally, it should be a period of shared care between CAMHS and AMHS, giving the patients the opportunity to understand the way the adult team works to properly prepare them for the change.

### It takes time to create a trusting relationship

In the current study, some patients had difficulty starting up a new relationship at AMHS with an open and trusting mind, and they supposed it would have been easier if they had received more support from CAMHS during the transition. The therapists’ approach was considered vital when establishing a therapeutic alliance as the patients’ social communication became more difficult when they felt unsafe. In accordance with our findings, previous studies have demonstrated that patients with AN are often particularly sensitive to other people’s opinions about them [32]. Similarly, Sly, Morgan [33] found that the patients’ first impressions of their new therapist often dictated if they would be open about themselves. They also found that young patients with AN needed a therapist that “talk[ed] straight” and required patients to be honest and open about their eating disordered behaviors; otherwise, the patients would disengage from the treatment.

The findings of this study underline the importance of the therapist’s timing to address sensitive subjects. The current study found that the participants wanted their therapists to take more time to get to know them before talking about the elements that triggered them most. A major focus on food, weight, and body from the start of the therapeutic relation made it difficult to trust their therapist. Previous research has underlined the importance of the therapist’s willingness to move slowly and collaborate on the therapeutic goals with the patients, especially in the earlier stages of therapy [34]. A trusting therapeutic relationship with patients with AN is considered a vital component of successful treatment, as these patients often are difficult to engage in treatment [16]. The participants often described how their ambivalent feelings toward recovery made it problematic to establish a new relationship with the therapists at AMHS. Ambivalence is described as one of the most challenging features of AN [35]. Swift and Greenberg [36] summarized 669 studies on dropout, and they found that studies with eating disorders, personality disorders, and higher proportions of young patients had higher dropout rates than other disorders. A stronger focus on building an alliance with the patient in these first meetings would influence the patients’ experience and their compliance with treatment. One issue that emerges from these findings is the complexity of clinical settings. Although the therapists acknowledge that establishing an alliance with patients is essential, they also have to meet expectations concerning efficiency, which gives them little time to do so [20].

### We are not all the same

In our study, the participants experienced the transition as an inflexible process. Some of the participants did not feel ready to be transferred to AMHS, but they had reached the age limit and were transferred anyway, which made them feel powerless. The participants viewed their developmental stage as more important for a successful transition than age. This is in line with findings of other studies conducted on transitions between CAMHS and AMHS [9, 17, 31] and in contrast to the clinical guidelines that guide therapists to collaborate with patients and relatives to find the optimal time for the transition [37]. The present study describes how the participants felt as though they were treated more as a patient than an individual when not included in the decisions made about their transition. This experience contrasts with the idea of the transition being a therapeutic process that empowers the patient’s recovery process [38].

As in our study, Cleverley, Lenters [31] underlined how the participants already are in a stressful situation and in need of a more individually adapted transition. Many participants thought that the transition would be better in the early 20s, when their lives were more stable. These results reflect some of the discussion about the term “adolescence.” The United Nations defines adolescence as the period of 10–19 years of age; however, Sawyer, Azzopardi [39] argued that this definition is outdated when considering contemporary patterns. They consider that extending the age group to 24 years corresponds more closely to adolescent growth and a contemporary understanding of this life phase. These ideas are consistent with other emerging literature on mental health transitions [40–42]. Raballo, Poletti [43] are concerned about the unwillingness to change the system to a more patient – oriented care that incorporates the full continuum of service response within a promotion and prevention framework for youth mental health. They are describing a more radical approach and suggest a substantial framework shift that reorient existing services towards youth- specific mental health care. With their proposition, the adolescent’s developmental stage and readiness would determine the timing of the transition. We assume that with such an approach the negative experiences described in the current study, would have been reduced.

### How they see me and treat me affects my hope for the future

The participants often described how they felt powerless, dependent on the system and the therapist’s impression of them, and were repeatedly defined as an “anorectic patient.” To maintain their motivation to recover, the participants needed to experience a therapist that perceived them as a person with unique qualities. Earlier research has described how patients with AN often feel treated as a diagnosis [44, 45]. Malson, Finn [46] found that both the patients and the therapists construct “the anorexic patient.” Defined by their disorder, everything the “patient” says is part of the AN, not the person. This resonates with our study, where participants expressed that this objectification of themselves influenced not only their self-worth negatively but also often their commitment to the treatment. These results reflect earlier studies’ claims that patients with AN associate negative treatment experiences with being treated as an anorexic, rather than an individual [47, 48].

One important finding in our study was how the participants emphasized the therapists’ use of words that deprived them of motivation and hope for a better future. Words such as “chronic” and “treatment resistant” made them lose hope for further treatment progress and the future. In a previous study, the interviewed professionals treating patients with AN were aware of how patients appraised and interpreted words about themselves or their condition. The professionals thus set high standards for how they communicated with the participants [20], and they felt under high pressure to do so from the patients and themselves. They also described a high level of anxiety and uncertainty concerning their assessments, and they elaborated on the consequences of potential premature termination of treatment. Empirical research suggests that therapists tend to develop more feelings of frustration, hopelessness, and helplessness in therapy with AN patients than with patients with other eating disorders [49]. However, existing studies tend to focus on patients’ characteristics when addressing dropout from treatment and fail to include systemic or therapist factors, implying that the high dropout rates are primarily the patients’ responsibility [36, 50]. Some issues that emerged from the findings of our study relate to the rapid turnover of therapists and how this affected how they attach themselves in relationships with their therapists and in private relationships. Swift and Greenberg [36] examined the premature discontinuation of adult psychotherapy and suggested that future therapeutic relationships can be seriously impeded by a repeated loss of therapists. One hypothesis is that when patients are forced into new relationships that rapidly terminate, it has a negative influence on their own and their therapist’s sense of achievement. This amplifies negatively both parties’ abilities to create a therapeutic environment and form a basis for a future trusting relationship.

### Strengths and limitations

The inclusion of experiences from a heterogenic patient group and the multi-perspective approach when analyzing the material may have provided validity to the study, otherwise missed [51–53] . This study has several limitations. The participants were a mix of self-selected and purposively selected participants, selected by a therapist or former therapist who viewed them as suitable for participating in the current study. The present study included only female participants; thus, a potentially different selection of participants may have emphasized other experiences. One obvious limitation is the retrospective nature of the interviews, which could represent a bias in material, as one often tends to remember the negative aspects better than the positive. The timespan of 0–10 years is wide; thus, participants could have been subject to recall biases. However, our study also had a thematic positive element. The participants connected meaningful elements from the transition to descriptions of consequences of the transition. Conducting qualitative interviews, the context as a phenomenon may influence the findings, here highlighted in two different ways. When trying to understand why and how particular experiences come into being, it can be a question of contextual factors. As we focus on patients’ experience, these are generated from different contexts and from different parts of the country and treatment facilities [54]. The interviews in the current study were conducted in different contexts and the researchers held different roles in each interview. It is likely to assume the researcher’s role as more active in the individual interviews compared to the focus group interviews. This, may have affected the contents of the interviews. At the same time, conducting individual interviews might also empower the participants by increasing their time frames to convey personal experiences without being influenced or modified by fellow participants.

## Conclusion

To conclude, it is of major importance to acknowledge the patients’ needs during the transition period and considering their readiness for the transition. To better the chances of a successful transition, both patient and relatives should be explained, in an explicit manner, the differences between the services. A stronger focus on building an alliance with the patient in the first meetings would influence the patients’ experience and their compliance with treatment. Taking into account the four dimensions described in the present study might improve the transition process and enhance the patients’ self-sufficiency and maturity.

## Data Availability

The datasets used and/or analyzed during the current study are available from the corresponding author upon reasonable request.

## Abbreviations

CAMHS: Child and adolescent mental health services
AMHS: Adult mental health services
AN: Anorexia Nervosa
OCD: Obsessive-compulsive disorder
PTSD: Post traumatic stress disorder
PCP: Primary Care Doctor
